# Brain/MINDS: brain-mapping project in Japan

**DOI:** 10.1098/rstb.2014.0310

**Published:** 2015-05-19

**Authors:** Hideyuki Okano, Atsushi Miyawaki, Kiyoto Kasai

**Affiliations:** 1 Laboratory for Marmoset Neural Architecture, Brain Science Institute RIKEN, 2-1 Hirosawa, Wako, Saitama 351-0198, Japan; 2 Laboratory for Cell Function Dynamics, Brain Science Institute RIKEN, 2-1 Hirosawa, Wako, Saitama 351-0198, Japan; 3 Department of Physiology, Keio University School of Medicine, 35 Shinanomachi, Shinjuku, Tokyo 160-8582, Japan; 4 Department of Neuropsychiatry, Graduate School of Medicine, University of Tokyo, 7-3-1 Hongo, Bunkyo-ku, Tokyo 113-8655, Japan

**Keywords:** brain mapping by integrated neurotechnologies for disease studies, transgenic non-human primates, optogenetics, tissue clearing, super-resolution microscopy, neuropsychiatric and neurodegenerative diseases

## Abstract

There is an emerging interest in brain-mapping projects in countries across the world, including the USA, Europe, Australia and China. In 2014, Japan started a brain-mapping project called Brain Mapping by Integrated Neurotechnologies for Disease Studies (Brain/MINDS). Brain/MINDS aims to map the structure and function of neuronal circuits to ultimately understand the vast complexity of the human brain, and takes advantage of a unique non-human primate animal model, the common marmoset (*Callithrix jacchus*). In Brain/MINDS, the RIKEN Brain Science Institute acts as a central institute. The objectives of Brain/MINDS can be categorized into the following three major subject areas: (i) structure and functional mapping of a non-human primate brain (the marmoset brain); (ii) development of innovative neurotechnologies for brain mapping; and (iii) human brain mapping; and clinical research. Brain/MINDS researchers are highly motivated to identify the neuronal circuits responsible for the phenotype of neurological and psychiatric disorders, and to understand the development of these devastating disorders through the integration of these three subject areas.

## 1. Introduction: brain-mapping projects

As happened for the human genome projects almost a quarter of a century ago, there is an emerging interest in brain-mapping projects as an international big science, including The Brain Research through Advancing Innovative Neurotechnologies (BRAIN) initiative in the USA and the Human Brain Project (HBP) in Europe (reviewed in [1]). In Japan, a brain-mapping project named Brain Mapping by Integrated Neurotechnologies for Disease Studies (Brain/MINDS) started in 2014 (http://www.brainminds.jp/) [2]. These three brain-mapping projects aim to reveal the structural and functional connectomics in the brain, and fundamentally contribute to the prevention, diagnosis and treatment of human brain diseases.

The USA and the European Union have different approaches toward brain mapping. The European Union HBP is a centralized, large-scale enterprise with a computational focus aimed at building detailed models of neural circuitry, along with 13 complementary sub-projects: SP1, Strategic Mouse Brain Data; SP2, Strategic Human Brain Data; SP3, Cognitive Architectures; SP4, Mathematical and Theoretical Foundations of Brain Research; SP5, Neuroinformatics; SP6, Brain Simulation; SP7, High-Performance Computing; SP8, Medical Informatics; SP9, Neuromorphic Computing; SP10, Neurorobotics; SP11, Applications; SP12, Ethics and Society; and SP13, Management (http://www.cordis.europa.eu/fp7/ict/programme/fet/flagship/). In the USA, the BRAIN Initiative is less centralized and is closer to traditional investigator-driven neuroscience research with different funding agencies (including the National Institutes of Health, National Science Foundation and Defence Advanced Research Projects Agency; http://www.whitehouse.gov/state-of-the-union-2013). An emphasis on the development of technologies to facilitate neuroscience research forms a basic theme for the BRAIN Initiative in the USA [2]. In addition to the BRAIN Initiative and the HBP, there is an emerging interest in brain-mapping projects in Australia, China and Japan.

## 2. Brain/MINDS in Japan

Research on the non-human primate brain is essential for understanding the human brain and for developing knowledge-based strategies for the diagnosis and treatment of psychiatric and neurological disorders. For these reasons, one of the important characteristics of Brain/MINDS is to devote considerable effort to mapping the brain of a small New World monkey, the common marmoset (*Callithrix jacchus*) [2,3]. The rationale for using a marmoset model rather than another animal model, including another non-human primate model, for human brain science was sevenfold: (i) as a primate, the marmoset brain shares some aspects of the developmental process and anatomical structure of the human brain; (ii) the marmoset has similar social behaviours to humans, including particularly a strong relationship between parents and offspring; (iii) the marmoset has a unique social vocal communication and there is a likely convergent evolution in this characteristic; (iv) there are neurological disease models of the marmoset that are comparative to human disease; (v) some of the higher cognitive tasks in marmosets are equivalent to those found in macaques; (vi) the marmoset can be handled with comparative ease owing to its small body size; and (vii) the marmoset has a strong reproductive efficiency. In addition, the common marmoset has the following advantages for brain mapping: (i) its frontal lobe is more developed and more similar to humans than that of other commonly used animals including rodents, which have limitations when trying to understand the human brain because of differences in the structure of the neocortex and neuronal circuits and behavioural paradigms; (ii) the brain is compact (approximately 8 g) and is suitable for comprehensive analysis of the neural circuits; (iii) marmosets are near-lissencephalic, making functional magnetic resonance imaging (fMRI), optical imaging, tracer injection and electrophysiological experiments easier; and (iv) marmosets can be genetically modified and manipulated [4–6].

In Japan, Brain/MINDS started in June 2014. To understand the higher brain mechanisms underlying human feelings and behaviours, researchers must integrate macro- and micro-level information from the whole brain. Through the development of novel cutting-edge technologies for brain imaging and manipulation, Brain/MINDS will use the marmoset model to reveal the structure and function of the brain, improve future diagnosis and treatment of psychiatric and neurological disorders, and establish new information technologies based on brain mechanisms.

The objectives of Brain/MINDS are categorized into three major subject areas, each undertaken by a separate group of researchers (see http://brainminds.jp) [2]:

(A) structure and functional mapping of the marmoset brain;
(B) development of innovative neurotechnologies for brain mapping; and
(C) human brain mapping and clinical research.

Brain/MINDS adopts both centralized and decentralized strategies. The researchers in Groups A and B belong to the RIKEN Brain Science Institute, which acts as a central institute, or to Keio University or Kyoto University, which act as affiliated institutes. The researchers in Group C are more distributed and are all involved in clinical neuroscience. The integration of these groups is essential for understanding human brain diseases from the standpoint of neuronal circuits. For example, in Brain/MINDS, researchers are highly motivated to identify the neuronal circuits responsible for disease phenotypes and reveal the causal relation between structural or functional damage of neuronal circuits and phenotypes of psychiatric disorders like schizophrenia.

## 3. Structure and functional mapping of the marmoset brain (Group A)

This research group is led by Hideyuki Okano (RIKEN Brain Science Institute and Keio University School of Medicine). The structural (anatomical) mapping of marmosets will be investigated at three different resolutions: macroscopic, mesoscopic and microscopic.

### (a) Macroscopic structural mapping

**Figure 1.**
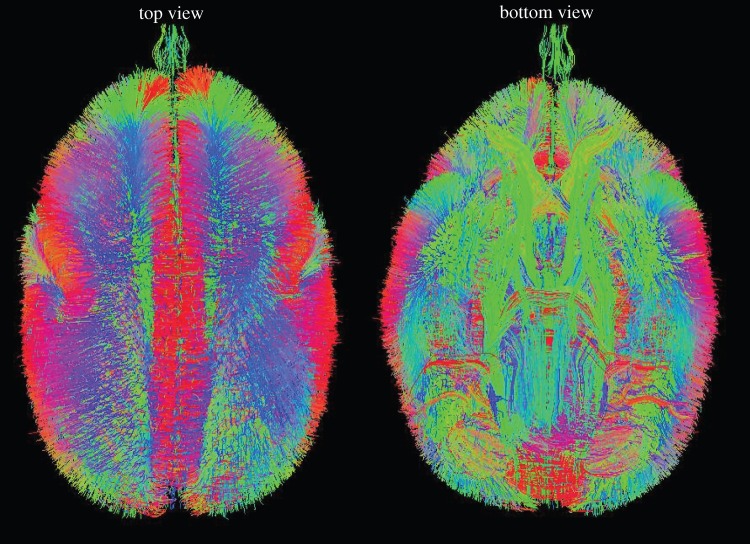
Reconstruction of whole-brain fibre structures of marmoset brain. Whole-brain tractography was reconstructed from high angular resolution diffusion MRI (HARDI) of marmoset brain and it enables analysis of the structural connectivity between remote anatomical regions in macro scale.

We will investigate the macroscopic structural map, particularly the inter-area map, of the marmoset brain using magnetic resonance imaging (MRI)-based diffusion tensor imaging (DTI) [2], which enables the tracking and visualization of neuronal fibres by taking advantage of the anisotropy of water molecule diffusion within the neuronal axons [7] (figure 1). Previously, corticospinal tracts [8], optic tracts [9] and the nigrostriatal pathway [10] of marmoset brains have been investigated using diffusion tensor tractography. In Brain/MINDS, DTI-based mapping with a high resolution (voxels of approx. 50 µm^3^ for *ex vivo* analysis and approx. 200 µm^3^ for *in vivo* analysis) will be developed and used [2]. DTI-based macroscopic mapping can be performed in a quantitative fashion together with voxel-based morphometric analysis, as already shown for a common marmoset model of Parkinson's disease [10]. We have investigated whether DTI can be used to detect the denervation of the nigrostriatal pathway in the marmoset model of Parkinson's disease and found that 1-methyl-4-phenyl-1,2,3,6-tetrahydropyridine-treated marmoset brains showed significantly increased axial and radial diffusivities in the bilateral nigrostriatal pathway, which is consistent with the observation that fibre structures of the nigrostriatal pathway were drastically decreased in the Parkinson's disease model. Thus, this study provides a potential basis for the use of DTI in the clinical diagnosis of Parkinson's disease. In 2011, we developed an MRI-based, tissue-segmented, population-averaged standard template of the common marmoset brain [11]. This template of the whole marmoset brain is available at the International Neuroinformatics Japan Node website (http://brainatlas.brain.riken.jp/marmoset/). The development of an MRI-based, population-averaged standard template enables us to examine voxel-wise statistics including voxel-based morphometry (VBM), which can be used to provide objective and bias-free information about brain structure and to detect differences in brain anatomy between a control group and an experimental group, such as a disease model, or to detect longitudinal changes within groups [12,13], such as ontogeny mapping of brain structure [2,5]. Currently, VBM is widely used in neuroanatomical study of various human mental or neurodevelopmental disorders including schizophrenia, drug-induced psychosis, autism and attention-deficit-hyperactivity disorder [13–17]. In Brain/MINDS, we will investigate the MRI-based macroscale mapping of various marmoset disease models with close collaboration with the researchers of Group C, who are involved in human brain mapping and clinical research.

### (b) Mesoscopic structural mapping

The mesoscopic scale of analysis, which employs light microscopy, is intermediate to the macroscale defined operationally by MRI/DTI and the microscale defined by electron-microscopic analysis. This scale of analysis aims to uncover the brain-wide connectivity and inter-area mapping by tracer injection and comprehensive mapping of gene expression by *in situ* hybridization. In gene expression mapping, we will focus on genes related to neurological and psychiatric disorders [18] or particular physiological functions that are well developed in primates, such as visual functions [19]. For the tracer injections, we will use the following methods. First, we aim to inject anterograde virus tracers into various parts of the marmoset brain, particularly the prefrontal cortex, using adeno-associated virus (AAV) vectors encoding three different fluorescent proteins (corresponding to green, red and blue colours) according to the marmoset brain in sterotaxic coordinates [20]. This work will be performed based on the methods described in a recent paper reporting a whole-brain dataset of anterograde injections in the mouse brain from the Allen Institute of Brain Research [21]. The stereotactic injections will be followed by systematic imaging of labelled axons by a high-throughput serial two-photon tomography system for constructing three-dimensional maps of the neuronal axons. In addition, Partha Mitra, a member of Brain/MINDS, will adopt a different approach by using anterograde and retrograde classical neuronal tracer injections on a systematic grid spanning the marmoset brain, and trace the axon tracts through the high-throughput Neuro Histology Pipeline in a similar way as in the Mouse Brain Architecture project (http://mouse.brainarchitecture.org). This work will be achieved collaboratively by the RIKEN Brain Science Institute in Japan and the Cold Spring Harbor Laboratory in the USA [2]. Transgenic techniques that can be used in the common marmoset [4,5], including genome editing [6], would enormously contribute to the mesoscale brain mapping aim of Brain/MINDS. We will generate transgenic marmoset lines that specifically express Cre-recombinase in particular neuronal subtypes, which are deeply involved in the pathogenic mechanisms of neurological or psychiatric disorders, such as dopaminergic neurons, parvalbumin-positive neurons, serotonergic neurons, glutamatergic neurons and cholinergic neurons. These Cre marmoset lines will be crossed with various reporter lines such as floxed-green fluorescent protein (GFP) for labelling neuronal axons, floxed-wheat germ agglutinin (WGA) for trans-synaptic tracing, floxed-GCamp for Ca imaging and floxed-channelrhodopsin 2 (ChR2) for optogenetics. Alternatively, these reporters can be introduced into particular sites within the marmoset brain through viral vectors (AAV vector or lentiviral vector).

### (c) Microscopic structural mapping

The Brain/MINDS project aims to map neural connections (connectomes) at nanometre resolution. This will be achieved using a new method of serial electron microscopy (EM) developed by Prof. Jeffery Lichtman's laboratory at Harvard University [2,22]. Serial EM is a recently developed technology that utilizes the scanning electron microscope to obtain serial images from continuous sectioning. The approaches involved in serial EM are widely applied to many projects, especially for analysing the three-dimensional microstructure of cells and tissues with high resolution. EM-based technologies enabled us to quantitatively map the precise location of cells, synapses and even organelles in a certain micro-domain of the brain [23–25]. Even though the marmoset brain is compact (approx. 8 g), determining the EM-based micro-connectome for the entire marmoset brain is not feasible within a limited period. Thus, we will focus on mapping the brain regions that are intimately involved in higher brain functions or disease-sensitive areas such as the prefrontal cortex, hippocampus and language-associated areas.

### (d) Functional mapping

To understand the working principle of the brain, it is essential to integrate structural and functional maps. In the Brain/MINDS project, we aim to perform fMRI-based mapping, positron emission tomography imaging and electrophysiological recording (including electrocorticography (ECoG) and multi-electrode recording) and Ca imaging using a miniature fluorescent microscope inserted within the brain in combination with an activity-dependent fluorescent reporter such a G-Camp [26]. fMRI-based functional connectivity has been studied through both task-based imaging and task-free imaging (resting state (rs)-fMRI) [27]. rs-fMRI is used for functional mapping of human brains in healthy subjects and patients with disease [27–29]. However, the relation between anatomical connectivity and functional connectivity speculated from rs-fMRI remains largely unsolved in human brains, and should be clarified in the current marmoset brain-mapping projects by the precise registration of rs-fMRI maps to DTI and other maps [2]. Task-based fMRI and positron emission tomography imaging will be used in the marmoset alongside the on-going development of behavioural tasks [30,31]. In Brain/MINDS, functional mapping will also be performed for marmoset disease models such as Alzheimer's disease models, Parkinson's disease/dementia with Lewy bodies models, psychiatric disease models and autism models that will be generated through the various transgenic technologies.

## 4. Development of innovative neurotechnologies for brain mapping (Group B)

This research group is led by Atsushi Miyawaki (RIKEN Brain Science Institute). Group B consists of the following three subgroups: (B1) development of techniques for high resolution, wide-field, deep, fast and long imaging of brain structures and functions; (B2) development of techniques for controlling neural activity; and (B3) development of neuroinformatics for integrating heterogeneous and multi-scale data.

Outstanding advances in genome science and gene technology have led to numerous discoveries and the development of new technologies in life sciences. The new technologies include optogenetics, tissue clearing and super-resolution microscopy. This technological innovation has been achieved thanks to the combined efforts of molecular biologists, electro-physiologists, brain anatomists and optical physicists. These technologies are becoming popular in neuroscience, where the central challenge is to understand the mechanisms by which neurons process and integrate synaptic inputs and how those mechanisms are modified by activity.

GFP was originally isolated from the light-emitting organ of the jellyfish *Aequorea victoria* in 1962. Thirty years passed before the complementary DNA-encoding protein was cloned in 1992 and subsequently characterized in 1994. Since this time, the ability of researchers to unravel the fine details of biological events has improved remarkably [32]. Furthermore, the emergence of the spectral variants of GFP, as well as GFP-like fluorescent proteins and chromogenic ligand-dependent fluorescent proteins from other organisms, has paved the way for researchers to simultaneously observe multiple biological events [33].

Optogenetic imaging with molecular sensors has great potential for investigations in neuroscience by virtue of its high spatial and temporal resolution. In the Brain/MINDS project, researchers have studied both the biological and practical aspects of various fluorescent proteins with the goal of enhancing their biological properties and making them practically useful. A large number of genetically encoded sensors have been developed for key intracellular environments or signalling molecules (events) [34], such as calcium ions (excitation) [35], membrane potential (excitation) [36], chloride ions (excitation) [37], pH (synaptic transmission) [38], glutamate (neurotransmitter release/uptake) [39], retinoic acid (metabolism) [40] and bilirubin (metabolism) [41]. These sensors can be used to investigate the function of specific signalling mechanisms in synaptic transmission, integration and plasticity, and to study neuronal firing inside the brain.

Optogenetic control of neuronal activity allows us to selectively activate or inactivate genetically defined populations of neurons to examine how the activity of neurons contributes to the function of neural circuits in the brain [42]. The light-activated ion channel ChR2 can be expressed in neurons, allowing brief flashes of blue light to activate the neurons. The expression of the light-driven chloride pump halorhodopsin allows for the inactivation of neuronal activity. However, many other important biological functions can also be controlled by light [43].

Such genetically encoded tools are introduced by gene transfer techniques into an intact organism and their expression is targeted to specific tissues, cell types or subcellular compartments, thereby allowing for the efficient detection or manipulation of neuronal activity. Owing to recent innovative progress in gene transfer techniques, including electroporation, viral-mediated gene transfer and germline transmission of transgenes, studies are no longer limited to mice but can also be performed in primates. The generation of transgenic marmoset lines (glowing monkeys) [4] inspired the Japan neuroscience community to launch the Brain/MINDS project.

The emergence of new tools stimulates the imagination of many neuroscientists. Light microscopes will inevitably have to be equipped with special hardware and software to maximize their use. In the Brain/MINDS project, researchers are developing light microscopy systems that are amenable to the addition of new functions for new technologies.

One important advantage of fluorescent proteins over organic chemical dyes is their ability to be genetically introduced into biological tissues regardless of the depth of the target area. With the advent of transgenic techniques to label specific cells with fluorescent proteins, life scientists are awaiting a new optical technique that can provide large scale and finely detailed perspectives of labelled structures within a large biological specimen. There is an increasing demand for new techniques that seek to address this issue, such as Brainbow mice [44]. Such techniques are critical for comprehensive connectomic analyses [45].

**Figure 2.**
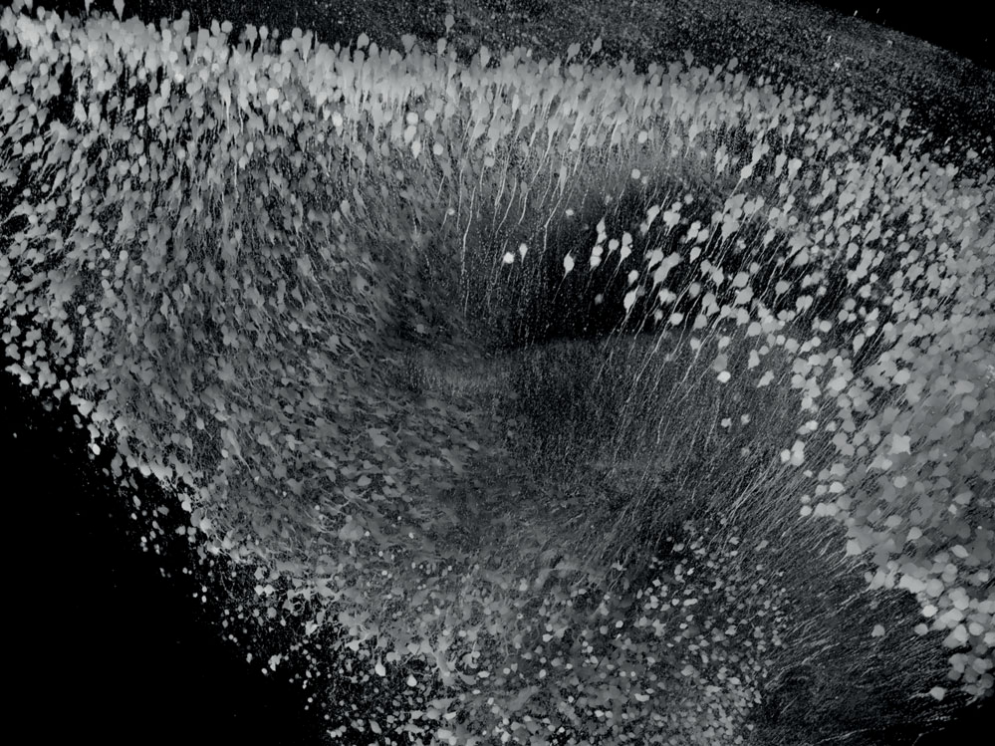
Clearing the mammalian brain. Three-dimensional reconstruction of yellow fluorescent protein- (YFP-)-expressing neurons in the hippocampal formation containing the dendate gyrus (DG) and Ammon's horn fields. The sample was excised from a fixed and optically cleared YFP-H mouse brain. Clearing was performed using ScaleA2 solution. This method will be applied for transgenic marmoset brains. (Copyright © RIKEN.)

The three-dimensional imaging of large biological specimens requires sectioning in order to improve axial resolution. It is also necessary to achieve subcellular resolution for the three-dimensional reconstruction of fluorescently labelled structures within large tissue samples, such as those from whole mouse brains. Mechanical sectioning methods allow for the efficient observation of genetically or immunohistochemically labelled structures with subcellular resolution, but involve extremely laborious three-dimensional reconstruction when performed on a large scale in the absence of well-designed automation procedures. Optical sectioning methods are highly promising, but optical imaging deep into tissue is prevented, mostly by light scattering. To overcome this problem, tissue-clearing technology aims to increase tissue transparency and achieve refractive uniformity throughout a fixed sample (figure 2). This technique involves the incubation of fixed brain samples in a clearing reagent for some time. Two types of tissue-clearing reagents are available: organic chemical-based solutions and aqueous solutions. Organic chemical-based solutions are highly capable of optically clearing fixed samples; however, the chemical clearing procedures substantially quench fluorescent proteins inside the samples. 3DISCO (three-dimensional imaging of solvent-cleared organs) was developed to solve this problem [46]. The 3DISCO procedure was then simplified to establish a simple, rapid and inexpensive method called iDISCO [47], which permits whole-mount immunolabelling with volume imaging of large cleared samples. A few aqueous solutions have been developed as tissue-clearing reagents, including FocusClear [48], Scale [49], ClearT [50], CLARITY [51], PACT (passive clarity technique) [52], SeeDB [53] and CUBIC [54]. In the Brain/MINDS project, we are improving these techniques to enable much larger scale three-dimensional imaging of molecularly labelled structures in cleared brain samples. Tissue clearing focuses on genetically expressed fluorescent markers, but should also be compatible with other labelling methodologies, such as immunohistochemistry. We also aim to prove the applicability of the clearing methods in tissue samples obtained from species that are not readily amenable to genetic modification, such as non-human primates and humans.

It is important to study the spatial regulation of a biological function within a sample at macroscopic, mesoscopic and microscopic levels. Although very few light microscopy techniques for three-dimensional reconstruction can penetrate tissue blocks thicker than 1 mm, most tomographic techniques, including optical projection tomography [55], computed tomography and positron emission tomography, as well as MRI, can analyse structural and quantitative features in much larger tissues, such as the whole body. Although current tissue-clearing techniques are limited to fixed biological samples, they are expected to enlarge the volume of three-dimensional reconstruction from light microscopy data, thereby bridging the imaging gap between the size of a specimen that can be visualized with light microscopy and the size of a specimen that can be visualized with other techniques. Likewise, the imaging gap between light microscopy and EM is also being reduced by strengthening the interactions between light microscopy and EM (correlative light microscopy/EM techniques) [56] or by increasing the spatial resolution of fluorescence imaging (super-resolution microscopy) [57], for which the Nobel Prize in Chemistry 2014 was awarded jointly to Eric Betzig, Stefan W. Hell and William E. Moerner.

In the third subgroup of Group B (B3), we plan to develop neuroinformatics for integrating heterogeneous and multi-scale data from microcircuit map, cortico-cortical projection, neural activity, and behaviour, with the aim of (i) the construction of a database and development of data analysis methods and (ii) multi-level data integration and large-scale model simulation. Notably, researchers in the B3 subgroup, including Yoko Yamaguchi, who is the head of the International Neuroinformatics Coordinating Facility (INCF) Japan Node, will improve the constructed databases through interactions within the Brain/MINDS project and will cooperate worldwide with the INCF, HBP, Allen Institute for Brain Science and the Kavli Foundation to generate refined databases with a common format. Basically, data sharing will be open source, with preference given to researchers who are interested in collaborating with the B3 group. Through these efforts, we will construct an atlas of the common marmoset brain by integrating heterogeneous big data. The data will be further used for multi-scale simulation to clarify the integrative principles of the marmoset and human brain using RIKEN's K Supercomputer.

## 5. Human brain mapping and clinical research (Group C)

This research group is led by Kiyoto Kasai (The University of Tokyo, Graduate School of Medicine). Here, we aim to map the brains of healthy control subjects and neuropsychiatric patients. Within Group C, the Clinical Research Organizing Team will organize three clinical research teams: the Psychiatric Disorders Research Team (Principal Investigator: K.K.), Neurodegenerative Diseases Research Team (PI: Hitoshi Okazawa, Tokyo Medical and Dental University) and Cerebrovascular and Neuro-rehabilitation Research Team (PI: Ryosuke Takahashi, Kyoto University). These clinical research teams will together generate a multi-centre database of patient MRI data and other biomarkers, and will provide feedback to marmoset researchers.

### (a) Background and goals

**Figure 3.**
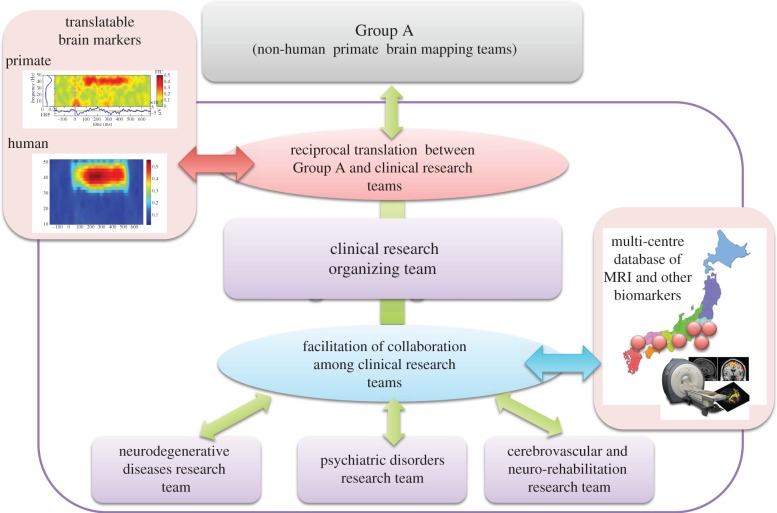
Research framework of Group C. Group C is responsible for human brain mapping and clinical research within Brain/MINDS. Through tight collaborations with Group A, the Clinical Research Organizing Team will organize the research conducted by Group C into three clinical research teams: Psychiatric Disorders Research Team, Neurodegenerative Disease Research Team and Cerebrovascular and Neuro-rehabilitation Research Team. Group C will manage a multi-centre database of structural MRI, rs fMRI and DTI data as well as data on other biomarkers to develop translatable brain markers that will facilitate reciprocal translation between human or clinical research in Group C and non-human primate research in Group A.

Disability-adjusted life years are an indicator of the impact of a disease on life and activities, and the number of disability-adjusted life years is larger for neuropsychiatric disorders than for diseases such as cancers and cardiovascular disorders. Neuropsychiatric disorders also represent a substantial financial burden on society; for example, the monetary cost of dementia in the United States was $100 billion in 2010 [58]. In medical research, the use of animal models is essential and effective for clarifying neurobiological mechanisms and screening drug discoveries. However, in research on neuropsychiatric disorders, particularly psychiatric disorders such as schizophrenia, rodent models have major limitations. The equivalence of behaviours and neurocircuits, particularly, those involving the prefrontal cortex, between humans and rodents cannot be assured. Thus, it has been a great challenge to identify molecular and circuit abnormalities and to develop pathophysiology-oriented intervention strategies through translation between basic and clinical research. The Brain/MINDS project uses marmosets, which are characterized by highly complex social behaviours and have a large prefrontal cortex (particularly lateral prefrontal cortex), which will be a major advantage for neuropsychiatric research over studies using rodents. Here, we propose a concept of a ‘translatable brain marker’, which refers to bridging the gap between human brain imaging and non-human primate brain imaging by using a measurement method common to both species (e.g. structural MRI, rs-fMRI, DTI, ECoG, electroencephalography (EEG), etc.) [59,60]. The main purpose of the clinical research teams is to develop translatable brain markers that are useful in research of neuropsychiatric disorders. This will be achieved by generating a large database of these markers in healthy individuals and in patients with neuropsychiatric disorders, and through tight communications with Groups A and B. The establishment of translatable brain markers will eventually lead to neurocircuit-based reclassification of neuropsychiatric disorders and neurocircuit-based biomarkers useful for clinical assessment and treatment. Standardization of the measurement protocol and acquisition parameters of the translatable brain markers will contribute to preclinical and clinical studies for drug discovery (figure 3).

### (b) Plans

The Clinical Research Organizing Team plans to (i) coordinate multi-centre collection of neuroimaging, neurophysiological and behavioural data in patients with various neuropsychiatric disorders to identify neural circuit abnormalities that are common across diseases and specific to a disease; and (ii) innovate technologies for human neuroimaging measurements and analysis. The neural circuit abnormalities identified in (i) will be translated to non-human primate studies led by Group A, and the technologies developed in (ii) will enable precise translation between marmoset brain maps and clinical neuroimaging and neurophysiological data.

The Psychiatric Disorders Research Team plans to (i) identify disease-related neural circuits by using neuroimaging, neurophysiological and behavioural data; (ii) develop psychiatry-oriented ‘translatable’ brain markers that can be measured using techniques common to humans and primates; (iii) characterize the clinical relevance of neurocircuits by circuit analysis and manipulation experiments using marmoset disease models; and (iv) establish neural circuit markers that can be used to reclassify diagnostic systems and to develop supplementary diagnostic tools and innovative treatment strategies for psychiatric disorders including schizophrenia, autism spectrum disorder, major depression and bipolar disorder.

We here describe a more detailed plan by illustrating neurophysiological studies in schizophrenia as an example. Previous structural MRI studies have shown a progressive decrease of neocortical grey matter volume in the early stages of schizophrenia that was coupled with an abnormality in a neurophysiological index of glutamatergic neurotransmission called auditory mismatch negativity [61,62]. Gamma-band frequency oscillations are thought be an index of gamma-aminobutyric acid (GABA)ergic neurotransmission and are abnormal in the early stages of psychosis [63]. Rodent model and human post-mortem studies have indicated that insult to dendritic spines through glutamatergic/GABAergic dysfunction may underlie the perionset period of progressive pathology in schizophrenia [64]. However, there has been no direct evidence of synaptic dysfunction in schizophrenia, a missing link between *in vivo* human, animal and post-mortem studies. To bridge this gap, neuroimaging and neurophysiological indices should be used to identify translatable brain markers that can be commonly measured in both humans and animals. Bidirectional animal and human research using translatable brain markers, such as markers from MRI and electrophysiology, will facilitate identification of effective molecular targets for early intervention for schizophrenia. Based on the hypothesis as described above, we plan to measure mismatch negativity and gamma-band oscillations in marmosets, macaques and in humans with and without psychiatric disorders including schizophrenia, by using EEG and/or ECoG where applicable.

The Neurodegenerative Diseases Research Team plans to detect the earliest change of neurocircuits in human neurodegenerative diseases including Alzheimer's disease, frontotemporal degeneration and diffuse Lewy body disease, to uncover the underlying molecular mechanisms and to develop therapeutics for human dementia by taking advantage of neurocircuit maps of transgenic marmoset models generated by Group A.

The Cerebrovascular and Neuro-rehabilitation Research Team plans to identify the injured and compensatory circuits that are present in patients with cerebrovascular disorders including motor paresis and higher brain dysfunction and Parkinson's disease. This team will develop new technologies to analyse the neurocircuits using rodent disease models, apply them to newly generated marmoset disease models, and establish translatable brain markers for neurocircuit injuries and subsequent recovery that are common to humans and animal models. These efforts will contribute to the development of new diagnostic tools based on circuit injuries and innovative therapeutics that accelerate the recovery of circuits in cerebrovascular disorders and Parkinson's disease.

Group C will deeply consider ethical issues associated with biomarkers and databases used in human studies. The Clinical Research Organizing Team will include experts in clinical ethics and will help researchers at each institution to obtain institutional review board approval. These experts will also monitor the appropriateness of measurements and the registration of biomarkers obtained from patients at each site as well as the accuracy of the database.

## 6. Conclusion and perspectives

Brain/MINDS is an ambitious project that aims to understand the higher brain mechanisms underlying human feelings and behaviours, to improve future diagnosis and treatment of psychiatric and neurological disorders and to establish new information technologies based on brain mechanisms. To better understand the human brain, we will take advantage of a non-human primate, the common marmoset. If we are able to obtain detailed information on the structural and functional connectivity of the entire marmoset brain, this will enormously contribute to our understanding of the human brain and its diseases [2]. Synthesis, mining and simulation of all datasets to understand human cognition and cure diseases are crucial for the success of the Brain/MINDS project.
